# Australian children living with rare diseases: experiences of diagnosis and perceived consequences of diagnostic delays

**DOI:** 10.1186/s13023-017-0622-4

**Published:** 2017-04-11

**Authors:** Yvonne Zurynski, Marie Deverell, Troy Dalkeith, Sandra Johnson, John Christodoulou, Helen Leonard, Elizabeth J Elliott, APSU Rare Diseases Impacts on Families Study group

**Affiliations:** 1 Australian Paediatric Surveillance Unit, Kids Research Institute, Westmead, NSW 2145 Australia; 2 Discipline of Child and Adolescent Health, Sydney Medical School, The University of Sydney, Sydney, NSW 2145 Australia; 3 Genetic Metabolic Disorders Service, Sydney Children’s Hospitals Network (Westmead), Westmead, NSW 2145 Australia; 4 Genetic Metabolic Disorders Research Unit, Western Sydney Genetics Program, Sydney Children’s Hospitals Network (Westmead), Westmead, NSW 2145 Australia; 5 Discipline of Child and Adolescent Health, and Discipline of Genetic Medicine, Sydney Medical School, The University of Sydney, Sydney, Australia; 6 Murdoch Children’s Research Institute and Victorian Clinical Genetics Services, Royal Children’s Hospital, Parkville, 3052 Australia; 7 Department of Paediatrics, University of Melbourne, Parkville, Vic 3010 Australia; 8 Telethon Kids Institute, The University of Western Australia, Crawley, WA 6009 Australia

**Keywords:** Rare diseases, Child, Families, Diagnosis, Diagnostic delays, Experiences, Australian

## Abstract

**Background:**

Children and families living with rare disease often experience significant health, psychosocial, economic burdens and diagnostic delays. Experiences appear to be constant, regardless of the specific rare disease diagnosis. Systematically collected Australian data to support policy response on rare diseases are scarce. We address this gap by providing survey results about 462 children aged <19 years living with approximately 200 different rare diseases.

**Results:**

Of 462 children, 96% were born in Australia, 55% were male, median age was 8.9 years (0–18.2). Four-hundred-and-twenty-eight (93%) had received a definitive diagnosis but 29 (7%) remained undiagnosed. Before receiving the correct diagnosis 38% consulted ≥ 6 different doctors. Among those with a diagnosis, 37% believed the diagnosis was delayed and 27% initially received a wrong diagnosis. Consequences of delayed diagnosis include anxiety, loss of reproductive confidence because of an ill-defined genetic risk, frustration and stress (54%), disease progression (37%), delays in treatment (25%) and inappropriate treatments (10%). Perceived reasons for diagnostic delays included lack of knowledge about the disease among health professionals (69.2%), lack of symptom awareness by the family (21.2%) and difficulties accessing tests (17.9%). Children with inborn errors of metabolism were less likely to have a delayed diagnosis compared with other disease groups (Chi-Sq = 17.1; *P* < 0.0001), most likely due to well-established and accessible biochemical screening processes. Diagnosis was given in person in 74% of cases, telephone in 18.5% and via a letter in 3.5%. Some families (16%) were dissatisfied with the way the diagnosis was delivered, citing lack of empathy and lack of information from health professionals. Psychological support at diagnosis was provided to 47.5%, but 86.2% believed that it should always be provided. Although 74.9% of parents believed that the diagnosis could have an impact on future family planning, only 44.8% received genetic counselling.

**Conclusion:**

Parents of children living with rare chronic and complex diseases have called for better education, resourcing of health professionals to prevent avoidable diagnostic delays, and to facilitate access to early interventions and treatments. Access to psychological support and genetic counselling should be available to all parents receiving a life-changing diagnosis for their child.

**Electronic supplementary material:**

The online version of this article (doi:10.1186/s13023-017-0622-4) contains supplementary material, which is available to authorized users.

## Background

Rare diseases are often complex, chronic, and cause intellectual and physical disability requiring frequent, ongoing access to multiple specialist health services [1–3]. Many have onset in childhood, but diagnostic delays are common, and treatment options limited [2, 4]. Although each rare disease is different, the challenges faced by families are often similar regardless of the specific diagnosis [2, 3, 5]. Prompt, correct diagnosis is very important for families, as it enables them to explain their child’s disease to others, to stop blaming themselves for their child’s condition, it may restore reproductive confidence and alleviates some of the stress of not knowing what is wrong and what to expect in the future. Delayed diagnosis or receiving the wrong diagnosis may lead to the use of inappropriate and potentially harmful treatments [6, 7]. Routine newborn screening for a number of rare disorders leads to prompt diagnosis, timely appropriate treatment and prevention of new cases [8], however, screening tests are available for very few of the ~8000 known genetic rare diseases. Lack of screening tests and limited knowledge among health professionals about how to recognise the signs and symptoms of rare diseases lead to diagnostic delays, and delays of 5 years or more have been reported among Australian adults [9].

For families, receiving a diagnosis of a rare chronic and complex disease for their child is often devastating and life-changing, and the diagnosis must be communicated with sensitivity in a supportive environment. In an Australian survey of adults, the diagnosis was delayed by five years or more in 30%, 66% saw three or more doctors to obtain a definitive diagnosis and many received at least one incorrect diagnosis [9]. Apart from some case studies, there are few large studies of the experience of parents receiving a diagnosis of a rare disease for their child [10–12]. In our study of 30 families of children diagnosed with inborn errors of metabolism (IEM), we found that the diagnosis was delayed in 43%, psychological support was seldom provided, and that 13% of parents were dissatisfied with the way the diagnosis was given [1]. Children living with rare diseases need frequent, multidisciplinary care, however, many find access to services difficult [1–3, 12]. Pelentsov et al. [13] recently published results from a survey of the experiences of caring for a child with a rare disease, which was completed by 285 Australian families in 2015 [13]. Parents felt socially isolated, anxious, fearful, angry, frustrated, and under-supported, while also expressing that access to healthcare was not equitable [13].

Significant diagnostic delays and inequitable access to diagnostic services for people living with a rare disease, prompted establishment of the Undiagnosed Diseases Program (UDP) in the United States of America (USA) in 2009 [7] and a similar program was established in Perth, Australia in 2015 [6]. Such programmes and the increasing integration of genomics into medical practice might reduce diagnostic delays and improve experiences for patients and families. Documenting current experiences of seeking and receiving a diagnosis is imperative to demonstrate the value of UDPs and genomic medicine for rare disease patients and their families. We describe experiences of seeking and receiving a diagnosis, and access to health care among 462 Australian families living with children affected by a large variety of rare diseases.

## Methods

The Australian Paediatric Surveillance Unit (APSU) Impact on Family Survey, developed, trialled and validated by us [1] was sent in 2013 to families identified as having children aged < 19 years and living with a rare disease. Families were recruited from lists held by our partner organisations:

1. The Steve Waugh Foundation (SWF), which provides financial support to families with children with rare diseases.
2. The SMILE Foundation (now part of Variety – the Children’s Charity), which provided emergency relief grants to families in financial crisis.
3. Genetic Alliance of Australia (formerly the Association of Genetic Supports of Australasia) which provides peer support and information for individuals and families affected by a genetic condition.
4. The Genetic Metabolic Disorders Service at the Sydney Children’s Hospitals Network (Westmead), a tertiary/quaternary children’s health facility.

The partner organisations were integral to the research team. They provided advice during survey development, assisted in developing the recruitment process, and carried out recruitment. Families were assigned a unique identifying code and the list linking identifying details with the codes remained with the partner organisation to protect privacy. The researchers received only the lists of codes. In our pilot study, families expressed a preference for completing the survey on paper rather than on-line [1]. Therefore, paper surveys and reply- paid envelopes were posted to 1761 families by our partner organisations on behalf of the research group. Families who did not respond within 3 months were followed up once by the relevant partner organisation.

The original survey [1] was slightly modified by adding questions about impacts on siblings and included the following sections: demographics, diagnosis, health functioning, treatment, health service use including barriers and enablers to access, impact on family including siblings, and need for psychosocial, economic, and peer support and information (Additional file 1). The family’s postcode enabled analysis of the Index of Relative Socio-economic Advantage and Disadvantage (IRSAD). IRSAD summarises information about the economic and social conditions of people and households within a geographical area [14]. We established whether children lived in urban, rural or remote regions of Australia according to The Accessibility/Remoteness Index of Australia (ARIA) [15].

This paper focuses on the demographics and diagnosis sections of the survey, and provides a description of the cohort, their experiences of diagnosis, diagnostic delays and perceived reasons for the delays, consequences of delayed diagnosis for the family, and level of satisfaction with the way the diagnosis was given. The survey was designed for completion by parents or carers who answered questions using multiple choice options, Likert scales, numerical answers e.g. age at diagnosis, and short text answers when opinions or further detail was needed.

### Data analysis

All categorical survey items were analysed using frequency distributions and cross tabulation. Continuous variables which were not normally distributed were analysed using non-parametric statistics including median and inter-quartile range. Text answers were scanned for themes and coded according to the themes identified by two scorers (YZ and MD) independently. The disease names provided by respondents were grouped into larger disease categories by an expert clinical geneticist (JC).

## Results

Of 1761 families invited to participate, 60 had children aged 19 years or more; 216 were not contactable by post, phone or email; and 20 had inadequate command of the English language to complete the survey, leaving 1465 potential respondents. A total of 462 (32%) surveys were completed and returned to the APSU. The survey was completed by the child’s mother (89%), father (8.5%) both parents (0.9%), foster carer (0.9%) or grandmother (0.2%). Thus 98.4% of respondents were parents and we refer to all respondents as parents throughout this paper.

### Demographics

The majority of children (70.1%) were from New South Wales (NSW) with smaller groups of respondents from other Australian states and territories. Compared with the distribution of children aged <19 years across other Australian states and territories, children from NSW were over-represented (Table 1). Among 462 children, there were 256 (55.4%) males, 206 (44.6%) females and their median age was 8.9 years (0–18.2 years), (Table 2). Almost all children (96%) were born in Australia. Other countries of birth included Afghanistan, America, Egypt, England, France, Iraq, Ireland, Israel, Lebanon, Malaysia, Philippines, New Zealand, Sri Lanka, Sweden and Ukraine. Seventy-eight per cent identified as Caucasian, 6% Middle-Eastern and 5% Asian (Table 2). Most children (84%) had at least one sibling. Approximately 60% of families resided in areas with an IRSAD score of 6–10, or relatively advantaged. According to ARIA codes [15], the majority (217, 75.5%) lived in capital cities, 67 (18.8%) in inner regional Australia, 25 (5.4%) in outer regional Australia, and 2 lived in remote or very remote Australia.

**Table 1:** Breakdown of our cohort by state/territory compared with proportion of the Australian population

State/Territory	Cohort N (%)	Population <19 years N (%)
New South Wales	324 (70)	1 780 756 (31)
Australian Capital Territory	10 (2)	89 739 (2)
Victoria	44 (10)	1 351 799 (24)
Queensland	51 (11)	1 180 313 (21)
South Australia	12 (3)	380 293 (7)
Western Australia	15 (3)	617 564 (11)
Tasmania	6 (1)	121 582 (2)
Northern Territory	Nil	66 739 (1)
Total	462	5 589 474

**Table 2:** Description of children living with rare diseases

Characteristics	N (%)
Gender	
Male	256 (55.4)
Female	206 (44.6)
Age Group (years)	
0 to 5	165 (36)
6 to 12	176 (38)
13 to 18	121 (26)
Country of Birth	
Australia	442 (96)
Other	20 (4)
Ethnicity	
Caucasian	360 (78)
Middle Eastern	28 (6)
Asian	22 (5)
Aboriginal or Torres Strait Islander	10 (2)
Diagnosis Groupings	
Inborn Error of Metabolism	176 (38)
Genetic Syndrome	63 (13)
Chromosomal Disorder	50 (11)
Congenital Malformation Syndromes	26 (6)
Neuromuscular Disorder	24 (5)
Gastrointestinal Disorder	10 (2)
Epilepsy Syndrome	9 (2)
Cardiac Disorder	7 (1)
Chromosomal Disorder/Genetic Syndrome	7 (1)
Neurodegenerative Disorder	6 (1)
Immune Disorder	6 (1)
Skeletal Dysplasia	6 (1)
Dermatological Disorder	5 (1)
Renal Disorder	5 (1)
Neurodevelopmental Disorder	3 (<1)
Respiratory Disorder	3 (<1)
Developmental Eye Disorder	2 (<1)
Skeletal Disorder	2 (<1)
Neuropathy	1 (<1)
Familial Cancer Disorder	1 (<1)
Endocrinological Disorder	1 (<1)
Connective Tissue Disorder	1 (<1)
Channelopathy	1 (<1)
Other^a^	16 (3)
Diagnosis Not Provided by family	5 (1)
No Diagnosis Yet	27 (6)

^a^ Other = rare disorders with <5 children in the sample, and combinations of diagnostic groups e.g. respiratory and neuromuscular disorder

### Diagnosis

Of the 462 children, 428 had received a definitive diagnosis including 17 children who had more than one rare disease; 29 were not yet diagnosed, and in five children the parents did not provide the diagnosis despite indicating that the child did have a diagnosis (Table 2). Over 200 different rare diseases were represented among the 428 children with a diagnosis. The most common disease group was IEM (38%), including medium chain acyl coenzyme A dehydrogenase deficiency (MCAD), phenylketonuria and galactosaemia. Genetic Syndromes (e.g. Noonan Syndrome, Rubenstein-Taybi Syndrome) accounted for 13% of diagnoses, and 11% had a chromosomal disorder (e.g. chromosome 2q 23.1 deletion, Jacobsen Syndrome, Phelan-McDermid Syndrome) (Table 2).

Seventy (16.4%) children had a relative with the same diagnosis, and in 22 individuals this was a first degree relative. Nine children had more than one relative affected by the same disease. In 7.2% the diagnosis was known before the child was born through antenatal imaging or genetic testing. In two such cases, the testing was done because a family member was affected.

Paediatricians, geneticists and neurologists were most often the first health professionals to raise the possibility of the diagnosis, and to confirm the final diagnosis, however, a wide range of health professionals were involved in diagnosing rare diseases (Table 3). The initial possibility of a diagnosis was sometimes raised by non-health professionals such as teachers, parents or relatives (Table 3). Half (51.7%) of the children were diagnosed after referral to a specialist clinic based in a large metropolitan paediatric hospital.

**Table 3:** Health professionals and others involved in diagnosis

Who initially raised the possibility of your child’s diagnosis?	N (%)	Who confirmed the final diagnosis for your child?	N (%)
Paediatrician	114 (26.6)	Geneticist	194 (45.3)
Geneticist	83 19.4)	Paediatrician	74 17.3)
Neurologist	43 (10.0)	Neurologist	54 (12.6)
Testing/newborn screening	24 (5.6)	Metabolic specialist	18 (4.2)
Nurse	20 (4.7)	Team of Specialists	11 (2.6)
General Practitioner	18 (4.2)	Cardiologist	8 (1.9)
Obstetrician	17 (4.0)	Gastroenterologist	5 (1.2)
Parent	17 (4.0)	General Practitioner	4 (0.9)
Neonatologist	9 (2.1)	Neonatologist	4 (0.9)
Team of Specialists	9 (2.1)	Nephrologist	4 (0.9)
Relative	7 (1.6)	Obstetrician	3 (0.7)
Metabolic specialist	7 (1.6)	Ophthalmologist	3 (0.7)
Allied Health Professional	7 (1.6)	Surgeon	3 (0.7)
Cardiologist	5 (1.2)	Haematologist	2 (0.5)
Gastroenterologist	5 (1.2)	Dermatologist	2 (0.5)
Teacher	4 (0.9)	Nurse	1 (0.2)
Nephrologist	3 (0.7)	Rheumatologist	1 (0.2)
Dermatologist	3 (0.7)	Radiologist	1 (0.2)
Haematologist	2 (0.5)	Oncologist	1 (0.2)
Ophthalmologist	2 (0.5)	Respiratory physician	1 (0.2)
Surgeon	2 (0.5)	Intensivist	1 (0.2)
Emergency Specialist	2 (0.5)	Immunologist	1 (0.2)
Rheumatologist	1 (0.2)	Hepatologist	1 (0.2)
Radiologist	1 (0.2)	Endocrinologist	1 (0.2)
Endocrinologist	1 (0.2)	Other^b^	15 (3.6)
Respiratory physician	1 (0.2)		
Anaesthetist	1 (0.2)		
Dentist	1 (0.2)		
Other^a^	7 (1.6)		
Total responses	420	Total responses	415

^a^Other included doctor or specialist not further specified, researcher or family friend

^b^Other includes doctor/professor not further specified including a doctor from overseas and “genetic testing”

### Diagnostic delays and consequences for families

Of the 428 diagnosed children, 179 (41.8%) consulted 3–5 different doctors, 71 (16.6%) consulted 6–10 different doctors, and 42 (11.1%) consulted more than 10 different doctors prior to receiving the definitive diagnosis. Most children (256, 59.8%) were diagnosed within the first 12 months of life, but 34 (8%) waited more than 3 years and one child waited 13.75 years. Across age groups, there was no significant difference in proportions of children whose parents reported diagnostic delays (Table 4). There was no association between socioeconomic status for area (IRSAD) or remoteness (ARIA) and delayed diagnosis (Table 4). Diagnostic delays were significantly less frequent among children diagnosed with IEM (30.2%) when compared with the other four most common diagnostic groups combined (54.6%) (Chi-sq = 17.1, *P* < 0.0001), (Table 4). Almost a third of children (117, 27.3%) had received a wrong diagnosis before receiving the correct definitive diagnosis. A second opinion to confirm the diagnosis was sought by 57 (13.3%) families. Over a third (157, 36.7%) of respondents believed that the diagnosis could have been made earlier. The perceived reasons for delayed diagnosis were provided by 156 respondents, and included lack of knowledge among health professionals (108, 69.2%), lack of family awareness of symptoms (33, 21.2%), delays in obtaining test results (30, 11.3%), lack of access to appropriate tests (28, 17.9%), and long waiting times to see doctors (16, 10.3%). Fifty-one (32.7%) respondents provided a great variety of other reasons for diagnostic delays including misinterpretation of ultrasound scans, concerns of parents being dismissed by health professionals, health professionals delaying genetic testing, tests not performed or inconclusive test results, test results were lost, the child has unusual signs and symptoms that could not be classified. The 157 children whose parents believed the diagnosis was delayed waited for a diagnosis significantly longer (median = 8.0 months, IQR 29.54) than children whose parents thought diagnosis was not delayed (median = 0 months, IQR 5.5).

**Table 4:** Diagnostic delays in the five most frequent rare disease groups in our sample

	N^a^	Perceived Delay N (%)	No Perceived Delay N (%)	Statistics
Age Groups				
0–4	126	52 (41.3)	74 (58.7)	Chi-sq = 0.53, *P* = 0.77
5–12	135	60 (44.4)	75 (55.6)	
13–18	98	45 (45.9)	53 (54.1)	
IRSAD				
Relatively disadvantaged (1^st^–5^th^ decile)	139	58 (42.0)	81 (58.0)	Chi-sq = 0.37, *P* = 0.54
Relatively advantaged (6^th^–10^th^ decile)	220	99 (45.0)	121 (55.0)	
ARIA				
Major Australian City	217	90 (41.5)	127 (58.5)	Chi-sq = 0, *P* = 1
Regional or remote Australia	65	27 (41.5)	38 (58.5)	
Diagnoses				
Inborn Errors of Metabolism	152	46 (30.3)	106 (69.7)	Chi-sq = 17.1, *P* < 0.0001
Four other common diagnostic groups combined	130	71 (54.6)	59 (45.4)	
Genetic Syndromes	50	23 (46.0)	27 (44.0)	
Chromosomal disorders	43	25 (58.1)	18 (41.9)	
Congenital malformation Syndromes	20	11 (55.0)	9 (45.0)	
Neuromuscular disorders	17	12 (70.6)	5 (29.4	

^a^Of the 462 families, 359 gave a definitive answer about delayed diagnosis; the rest either did not answer or ticked “don’t know”

Of the 157 respondents who believed that diagnosis was delayed, 64 provided comments about the consequences for their families and 28 (43.7%) provided more than one consequence. Of the 64 providing comments, anxiety, frustration and stress were the most common consequences (54.7%), followed by worsening of symptoms and disease progression (37.5%), delays in treatment or early intervention (25.0%), use of inappropriate treatments (10.9%), additional medical costs (9.4%) and other consequences including impacts on family relationships and siblings (18.8%). Twenty-four (15.2%) respondents indicated that the diagnostic delays led to extra diagnostic tests.

### Satisfaction with the way diagnosis was given and psychological support

Psychological support was offered to 201 (47.5%) of families around the time of diagnosis and was provided by a social worker in 46.8% cases, a genetic counsellor in 45.3%, and a specialist doctor in 27.9%, with some receiving support from more than one health professional. Others providing support included general practitioners, psychologists, peer support group members, nurses and case managers. Three hundred and sixty-nine (86.2%) respondents believed psychological support should always be offered to families at the time of diagnosis. Over a half of respondents (57.7%) believed that receiving the diagnosis for their child would have an impact on future family planning, but only 44.8% received genetic counselling.

**Fig. 1:**
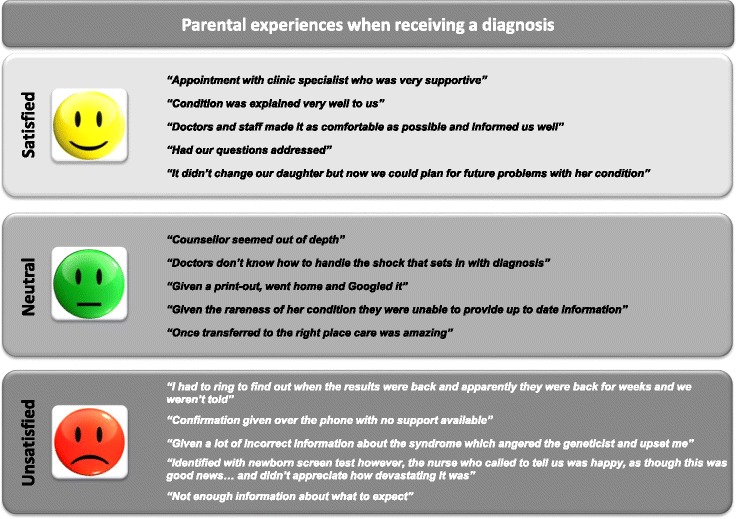
Illustrative comments about experiences of the way diagnosis was given to the family

Of the 428 children who had a diagnosis, the diagnosis was given in person in 74.3% cases, via telephone in 18.5% and via a letter in 3.5%. Most respondents (65.9%) were very satisfied or satisfied with the way in which they were told about their child’s diagnosis but 18.0% were neutral and 16.1% were dissatisfied or very dissatisfied. Illustrative examples of comments provided by families are summarised in Fig. 1. The most common issues identified among families who were dissatisfied included: lack of empathy, lack of information or wrong information provided about the disease, and avoidable delays (Fig. 1).

Parents who were satisfied with the way diagnosis was given were less likely to have experienced diagnostic delays (Chi-sq = 7.84, *P* < 0.05) and were more likely to have been offered psychological support (Chi-sq = 6.30, *P* < 0.05).

### Children without a diagnosis

Twenty-nine (6.3%) of children had not received a diagnosis at the time of completing the survey. Their ages ranged from 2 months to 18 years (median 8.06 years) and the median age at the onset of symptoms was approximately 2 weeks (range: birth to 4 years). At the time of completing the survey, these children had already waited for a diagnosis for 6.4 years (median). The median waiting time for the five undiagnosed children aged 13–18 years was 13.75 (range: 13.5–15.6) years, for those aged 5–12 years it was 8.6 (range: 5.1–12.3) years and for the 0–4 year old children it was 3.2 years (range: 3 months–4.6 years). The majority identified as Caucasian and all spoke English at home; two of the children were born overseas, one in England and one in Sweden. In their search for a diagnosis (41.4%) of these families had already consulted 3–5 different doctors, 24.1% consulted 6–10 different doctors and (11.1%) consulted more than 10 doctors.

## Discussion

In agreement with previous studies, parents reported that delayed diagnosis had significant impacts on themselves, their child and family, including anxiety, frustration and stress, worsening symptoms or disease progression, delays in treatment or early intervention and use of inappropriate treatments [1–3, 5, 10, 11, 16]. Of children, 8% had waited over 3 years for a diagnosis. Long diagnostic delays were also reported among Australian adults [9] and among children and adults living in Europe and the United Kingdom (UK) [5, 17]. Parents of children diagnosed with IEM in our study were significantly less likely to report diagnostic delays. This is because newborn screening programmes incorporating accessible biochemical tests are integrated into the Australian health system where all newborns are offered screening for amino acid disorders including phenylketonuria, organic acidaemias, and fatty acid oxidation defects and because of access to specialised clinics for IEM [8].

The most common perceived reasons for delayed diagnosis reported by parents participating in our study was the lack of knowledge among health professionals. Lack of knowledge among health professionals was also described as a leading cause of diagnostic delays in the Australian survey of adults living with rare disease [9]. Parents reported that diagnostic delays had serious consequences for them and their child including stress, worry and frustration. A recent study of experiences of caring for children with a rare disease also showed high levels of stress and anxiety among parents, and this was associated with a perceived lack of knowledge about rare diseases among health professionals [13]. Other perceived serious consequences of delayed diagnosis in our study included worsening symptoms and disease progression, delays in accessing treatments and early intervention programmes, and wrong medications being given.

Receiving a diagnosis of a rare chronic and complex disease for their child is a life-changing event for many families, and most require support at or near the time that diagnosis is made. Almost all parents in our study believed that psychological support should always be offered at the time of diagnosis, but less than half of them received such support. Furthermore, fewer than half of the families who had a child with a rare genetic disease, which they believed would have consequences for future family planning, had received genetic counselling. For families planning future pregnancy, this information is of paramount importance to enable them to make informed choices about their reproductive options, thereby restoring reproductive confidence. The Australian health care system needs to increase the capacity for psychological and genetic counselling by embedding psychologist and genetic counsellors in all services where the diseases are diagnosed.

Most families were satisfied with the way that diagnosis was given, however 16% were dissatisfied, citing reasons such as inadequate information about the disease being provided, lack of psychological support, and lack of sensitivity among health professionals when giving the diagnosis. This was summed up by one respondent who said that the health professional giving the diagnosis: “…didn’t appreciate how devastating it was”. Our results concur with the EURORDIS Care 3 survey conducted by the European Organisation for Rare Diseases in 2010 which also showed that in 23% of patients the diagnosis was announced inappropriately, and for 12% it was done in an “unacceptable” way [5]. Training and resources are needed to up-skill health professionals to enable them to give significant, life-changing news in an appropriate and sensitive manner. Health service planners and providers should consider strategies for increasing the capacity for provision of psychological support for all families who have recently received or are soon to receive a diagnosis of a rare chronic and complex disease for their child.

Reaching a definitive diagnosis in patients with rare diseases is challenging for health professionals [2–4]. Although paediatricians, geneticists and neurologists most commonly diagnosed rare diseases in our study, a great variety of medical specialists and generalists were also involved, and all health professionals should have some training about rare diseases. At the very least, health professionals need to know where to find information about rare diseases, including how to access respected websites such as Orphanet and Online Mendelian Inheritance in Man for current, evidence-based information to inform their discussions with families [18, 19].

The difficulties in reaching a diagnosis in children with rare diseases were illustrated by the 29 children who were undiagnosed at the time of our survey. In all of these children the symptoms had started before two months of age, and there were five children who had waited between 13 and 18 years for a diagnosis but remained undiagnosed. Advances in genomics and improved referral and interdisciplinary approaches, including through UDPs, has resulted in some patients with undiagnosed syndromes receiving a diagnosis. Moreover, it has recently been shown that early introduction of diagnostic whole exome sequencing in children with so-called “childhood syndromes” is far superior to the current standard of care diagnostic processes [20]. Currently there is no data about the experiences of patients who receive a diagnosis through UDPs. Often these services require that all avenues for diagnosis are exhausted before accepting patients, and this process itself may delay diagnosis. Despite the USA UDP being active since 2008, it is only recently that patient experiences, including access to health services, treatments and psychosocial impacts have been raised as important outcomes [21]. We believe that any UDP should integrate an evaluation which seeks to assess patients’ and families’ experiences while interacting with the UDP, before and after they are discharged from the UDP with or without a diagnosis. Furthermore, it is not clear what supports are provided to patients who are discharged from a UDP without receiving a definitive diagnosis. Such patients may need significant ongoing psychological support as they face the possibility that their child’s disorder may never be diagnosed. Peer support groups established specifically to support undiagnosed patients e.g. Syndromes Without A Name (SWAN) may meet the needs of such patients [22].

The over-representation of children and families from NSW is a limitation of our study; however, our cohort includes children from all states and territories except for the Northern Territory where the population of children is approximately 1% of Australia’s total. The over-representation of children with IEM reflects recruitment from the largest IEM service in Australia located in NSW. The peer support organisations used to recruit children/families into our survey have national scope, however, all of these organisations are physically based in Sydney NSW, and this is likely to have skewed recruitment. Strengths include recruitment of a large sample of Australian children/families which compares favourably with samples recruited in regions with much larger populations than Australia (23 million) e.g. EURORDIS Care 3 survey (*N* = 5995, response rate 30%; population 742 million) [5] and the surveys conducted by Rare Diseases UK (*N* = 570 in 2010; *N* = 1203 in 2016; no response fraction calculated; population 64 million) [17, 23]. The large variety (over 200), rare diseases represented among our cohort is a strength because our results are likely to be generalizable to children living with other rare diseases and the issues faced by families are similar regardless of the specific diagnosis [2–4, 24–26]. The greatest strength of our research was the direct involvement of peer support organisations. They provided important insights to improve the relevance of the survey to families.

We were able to calculate a response fraction (32%) and to assess representativeness, and we are also confident that the surveys returned were completed by each respondent only once. This cannot be confirmed for studies that used on-line surveys open for anyone to complete [9, 13]. Although a response fraction of 32% is in keeping with other studies, families who had bad experiences might have been more motivated to participate, and might have introduced a bias. A potential limitation may be that the survey was de-identified, precluding any verification of the child’s diagnosis from medical records. However, parents of children with rare diseases have been shown to be very knowledgeable about their child’s disease [5, 17].

## Conclusions

Our study establishes the largest systematically recruited Australian dataset of the experiences of parents whose child received a rare disease diagnosis. This paper describes only a small fraction of the data collected in the survey and other publications about the psychosocial and economic impacts of rare diseases on families are planned. Our findings suggest that psychological support is desired by almost all parents whose child receives a rare disease diagnosis, and should be routinely offered. Parents believe that health professionals’ knowledge about rare diseases needs to improve to enable more timely diagnosis, treatment, and provision of accurate information about the implications of the disease to families who are stressed, frustrated and anxious. The integration of genomic medicine into the health system, the establishment of multidisciplinary specialist clinics, and clear referral pathways may improve the timeliness and accuracy of diagnosis for children with rare diseases. The ultimate aim should be to improve patient and family experiences, and it is therefore imperative that patients are involved in development and evaluation of such programs.

## Additional file

Additional file 1: APSU Impact on Family Survey. (PDF 703 kb)

## Abbreviations

APSU: Australian Paediatric Surveillance Unit
ARIA: Accessibility/Remoteness Index of Australia
IRSAD: Index of Relative Socio-economic Advantage and Disadvantage
IEM: Inborn Errors of Metabolism
MCAD: Medium-chain acyl-coenzyme A dehydrogenase
SWAN: Syndromes Without A Name
SWF: Steve Waugh Foundation
UDP: Undiagnosed Diseases Program
UK: United Kingdom
USA: United States of America
