# Supplementary material for: Australian children living with rare diseases: experiences of diagnosis and perceived consequences of diagnostic delays
Source: Orphanet J Rare Dis. 2017 Apr 11;12:68. doi: 10.1186/s13023-017-0622-4 (PMC5387276; doi:10.1186/s13023-017-0622-4)
Supplement: Additional file 1: — APSU Impact on Family Survey. (PDF 703 kb) [file 13023_2017_622_MOESM1_ESM.pdf]

Living with Rare Disease:
The Impacts on your Child and your Family

INFORMATION STATEMENT

We would like to invite you to be a part of a project that looks at the experiences of families like yours, who are caring for a child with a rare disease or condition. There are approximately 8000 different rare diseases affecting an estimated 2 million Australians, including 400,000 Australian children. Approximately 70% of all rare diseases are of genetic origin; however, some are due to rare infections, injuries or mental health problems.

What is the project about?

For the first time in Australia, this project brings together a number of rare disease charitable organisations, to work with a nationally recognised research partner at the University of Sydney, the Australian Paediatric Surveillance Unit (APSU), to adopt a coordinated approach to the provision of better support services, educational opportunities, and information resources for families of children with rare diseases. This approach will greatly increase the awareness of rare diseases amongst the public and government sectors and provide a knowledge base about rare diseases for future research in this neglected area.

We have developed a survey for parents/carers of children with rare diseases to explore your experiences during the diagnosis period, the health related function of your child, the impact that caring for your child has had on your family and your experiences with Health and Support Services.

What will it involve?

You will be asked to complete a survey about your child(ren) **aged less than 19 years** with a rare disease/condition and your family and return it in a postage paid envelope. The survey will take about 1 hour to complete.

What will happen to the information collected?

All information you provide is confidential.

The information we collect will be anonymous, it will not contain your name, your child's name or contact details. Each survey will have a code attached that will be linked with each family. Only our organisation will have access to that code which will be kept in a secure database. Only the research team collecting the data will have access to your questionnaire answers but not any identifying information about you. All survey responses will be entered onto a computer protected by a password in a locked office in a secure building at the Children's Hospital at Westmead. Data from the study will be analysed and published, but will not contain any data which could identify individuals, including your child or your family. Only summary data will be published.

Feedback will be provided to you through our newsletters informing you of results and how they will be used to inform improvements to support services, educational opportunities and information resources for families such as yours.

Do I have to take part in the study?

Participation in this survey is completely voluntary. If you do decide to be a part of this project, by completing the survey, you will be giving consent for us to use the anonymous information you have provided.

You are able to withdraw from the study at any time and this will not affect your relationship with any of the partner organisations in any way.

Are there any concerns you have with the study?

If you have any concerns or issues regarding this study or questions asked in the survey please contact the APSU on 02 9845 3005 or the Social Work Department at the Children's Hospital at Westmead on 02 9845 2608.

Other information

If you have any questions, feel free to contact us at any time.

If you have specific questions about the survey or the design of this research, please contact:

Dr Marie Deverell marie.deverell@health.nsw.gov.au or phone the Australian Paediatric Surveillance Unit on
02 9845 3005.

This research study has been approved by the Sydney Children's Hospitals Network, Human Research Ethics Committee (HREC reference number 10/CHW/75).

Australian Government
Australian Research Council
This research is supported under the Australian Research Council's Linkage Projects funding scheme (project number LP110200277)

Living with Rare Disease:

The Impacts on your Child and your Family

Family code…………….

Living with Rare Disease:

The Impacts on your Child and your Family

INSTRUCTIONS FOR COMPLETION OF THE SURVEY

Thank you for your interest in taking part in this research project.

By completing and returning this survey you give consent to participate in this study, and so it is important that you understand what is required and how the information you provide will be treated and used. For this reason ***it is important that you read the information and instructions on this sheet carefully*** before deciding to complete and return the survey. If you have any questions please do not hesitate to contact the APSU (Phone: 02 9845 3005) or Dr Marie Deverell marie.deverell@health.nsw.gov.au.

How to complete this survey

This survey has 15 pages and 8 sections:

- About you and your child
- Diagnosis
- Health Related Function
- Treatment
- Health Service Use
- Impact on Family
- Support and Information Needs
- Financial Support.

The questions concern you and your experiences whilst caring for your child or children who have a rare disease. You will need to complete a separate survey for each of your children as each child's transition journey will be different.

We estimate that it will take approximately one hour to complete the questionnaire. Please note that you need not complete it in one session.

Some questions will simply require you to choose an answer out of options we have provided by marking a box with an **X** or tick (√), others will ask you to fill in the space provided by typing or writing.

Try to select the most appropriate answer to each question. In some instances you may not know the answer, so please tick the 'Don't Know' (DK) option. Some questions will ask you to complete them only if you answered yes to the question before it. Please follow the directions in the survey to ensure that you answer all required sections.

Please remember all the data is anonymous, so **please *DO NOT*** *write your name, address, phone number or any other information that might identify you on this survey.* Some questions ask about the diagnosis, how this was made and who made it. *To protect the privacy of the health professional(s) who might have been involved in the diagnosis*, ***please DO NOT*** *provide any information that might identify them (e.g. DO NOT provide any health professionals' names, phone numbers or places of work).*

Confidential

ABOUT YOU AND YOUR CHILD

Instructions: *Answer for the first affected child living in your household. Answer each question by ticking the appropriate box or writing your response in the space provided.*

DK= Don't Know, NA = Not applicable

1. Who is completing this questionnaire?

☐ Mother ☐ Father ☐ Foster carer
☐ Other (specify) ______________________________

2. How old is your child with the rare disease? Years: ______________Months:______________

3. What is your child's gender?

☐ Male ☐ Female

4. What is the postcode of your current residence? ☐☐☐☐

5. In which country was your child born? ______________________________

6. Which of the following would best describe your child's ethnicity?

☐ Caucasian ☐ Aboriginal ☐ Torres Strait Islander ☐ Both Aboriginal and Torres Strait Islander
☐ Asian ☐ Maori ☐ Pacific Islander ☐ Middle Eastern
☐ African ☐ Other (specify) ______________________________

7. What is the primary language spoken at home?______________________________

8. Has your child received a diagnosis?

☐ Yes. Which rare disease does your child have? ______________________________
☐ No
If No, please answer a) and b) below and then go to ***question 27*** on page 4

a) How old was your child when the symptoms or signs of disease first started?

Years:______________ Months:______________

b) How many doctors (approximately) were consulted about your child's first symptoms or signs of disease?

☐1 - 2 ☐ 3 - 5 ☐ 6 - 10 ☐ 11 - 20 ☐ >20

9. Has anyone else in the family ever had this disease?

☐ Yes, specify relationship/s to the child (e.g. sister, etc):______________________________
☐ No ☐ Don't know

DIAGNOSIS

10. When was your child diagnosed?

☐ Before birth (specify circumstances, e.g. screening because of other cases in the family, routine screening, maternal illness during pregnancy, etc):

____

____

☐ After birth (specify age): Years: ______________Months:______________

11. If diagnosed after birth, how old was your child when the symptoms or signs of the disease first started?

Years: ______________Months:______________ ☐No signs/symptoms

Confidential

12. How many doctors (approximately) were consulted between first symptoms or signs of disease and the final diagnosis?

☐1 – 2 ☐ 3 – 5 ☐ 6 – 10 ☐ 11 - 20 ☐ >20

13. Was your child given any other diagnoses before the final diagnosis was made?

☐ Yes ☐ No ☐ Don't know

a) ***If yes***, please list all previous diagnoses given

____

b) ***If yes,*** do you believe this led to any unnecessary tests or treatment?

☐ Yes ☐ No ☐ Don't know

14. Who initially raised the possibility of your child's diagnosis?

☐ Geneticist ☐ Neurologist ☐ Paediatrician ☐ Obstetrician
☐ GP ☐ Relative ☐ Teacher
☐ Other (including other specialist, etc):______________________________

15. Who made the final diagnosis?

☐ Geneticist ☐ Neurologist ☐ Paediatrician ☐ Obstetrician
☐ GP ☐ Relative ☐ Teacher
☐ Other (including other specialist, etc):______________________________

16. Was the diagnosis made in a clinic specialising in your child's disease?

☐ Yes ☐ No ☐ Don't know

If yes, what was the setting for the clinic:

☐ Large metropolitan Children's Hospital
☐ Other hospital (please specify) ______________________________
☐ Specialist (private rooms)
☐ Other:______________________________

If yes, what led you to/how did you find this clinic?

☐ Recommended/referred by doctor
☐ Recommended/referred by another health professional
☐ Internet
☐ Recommended by another patient or family
☐ Other (specify): ______________________________

If yes, where was this clinic in relation to your home at the time?

☐ Same city ☐ Same state ☐ Same country ☐ Another country

17. How were you told about the diagnosis?

☐ Orally, if yes, please specify where (consultation room, corridor, waiting room):______________
☐ By phone ☐ In writing, with explanations ☐ In writing without explanations
☐ Other (specify): ______________________________

18. Did you seek a second opinion to confirm the diagnosis?

☐ Yes ☐ No

19. Do you believe that the diagnosis of your child's rare disease could have been made earlier?

☐Yes ☐No ☐Don't know

a) If yes, why do you think the delay occurred? (P*lease tick all that apply)*

☐ Lack of knowledge among health professionals about the disease
☐ Tests not available for the disease
☐ Tests took a long time
☐ Long waiting period to see specialist
☐ Lack of family awareness at early stages
☐ Other (specify) ____________________

b) If yes, please state what (if any) consequences the delay had for you and your family:

____

____

20. Did your child's diagnosis lead you to move closer to medical facilities? ☐ Yes ☐ No

a) If yes, specify:

☐ Within same city ☐ Within same state ☐ Another state ☐ Another country

21. Were you offered counselling or psychological support at the time of diagnosis? ☐ Yes ☐ No

a) If yes, specify by whom:

☐ GP ☐ Specialist ☐ Genetic counsellor ☐ Psychologist
☐ Social worker ☐ Support group member
☐ Other ____________________

b) If yes, was ongoing counselling or psychological support offered? ☐ Yes ☐ No

22. Do you believe that counselling or psychological support should always be offered at the time of diagnosis?

☐ Yes ☐ No ☐ Don't know

23. What information would you like to be given at the time of diagnosis? *(Please tick all that apply):*

☐ Information about the disease and what causes it
☐ Support organisations related to the disease
☐ How the diagnosis will affect your child's daily life
☐ Prognosis or what to expect in the future
☐ Specialist medical centres related to the disease
☐ Respite care options

24. How would you like to receive this information? *(Please tick all that apply):*

☐ Written / pamphlet ☐ Orally ☐ Website reference
☐ Other: ____________________

25. Is your child's disease/condition genetic in origin? ☐ Yes ☐ No ☐ Don't know

If yes, will this have an impact on future family planning? ☐ Yes ☐ No ☐ Don't know ☐ N/A

If yes, have you been offered genetic counselling? ☐ Yes ☐ No ☐ Don't know

26. Overall, how satisfied were you with the way in which you were told of your child's final diagnosis?

☐ Very satisfied ☐ Satisfied ☐ Neutral ☐ Not satisfied ☐ Very unsatisfied

Please provide a brief statement on the reasons for your answer to the above question:

____

____

Confidential

HEALTH FUNCTIONING

27. Please describe your child's health functioning according to the scale below:

*Tick the box which BEST describes how your child is **NOW**, compared with other children of the same age. Don't worry if some parts of the description do not apply to your child. Just choose the **BEST description**.*

CATEGORY	TICK ONE ONLY	DESCRIPTION TO HELP
**Superior**	[ ]	No symptoms; physically able; excellent relationships with family and friends; wide range of extra-curricular activities; doing well at school/preschool; developing normally; everyday problems never get out of hand.
**Good in all areas**	[ ]	Virtually no symptoms; usually copes well; physically able; good relationships; normal play & leisure activities; school/preschool OK; may have problems when stressed but these are short lived and only occasionally get out of hand.
**No more than slight problems**	[ ]	Some significant symptoms, only briefly get out of hand; sometimes child gets distressed; short term or little interference with mobility or relationships or play & leisure activities; school/preschool may be slightly affected or affected for a short time.
**Some difficulty in a single area but generally pretty well**	[ ]	Mild symptoms which recover quickly with treatment; any distress or disability does not stop child from doing most things at that age; some anxiety or irritability or brief mood changes; minor effect on mobility or school/preschool or relationships or play & leisure activities; problems may persist but may only be recognized by those who know the child.
**Variable problems in some but not all areas**	[ ]	Moderate symptoms have significant disabling effect on child; minor to moderate effect on mobility; school/preschool may be affected; may need special education; in some situations may seem O.K.; mainly managed in outpatient clinic or family doctor.
**Severe problems in one area OR moderate problems in most areas**	[ ]	Severe symptoms having a major effect on child's life; restricted mobility; relationships or play & leisure activities are affected; child is distressed or has difficult behavior; some relationships are maintained; learning difficulties or problems with or missing school; likely to have been seen by specialist.
**Major problems in several areas AND unable to function in one of these areas.**	[ ]	Severe, almost constant symptoms; child is distressed, withdrawn or has strange or aggressive behaviour; significant limitations on mobility or school/preschool or relationships or play & leisure activities; specialist management needed.
**Unable to function in almost all areas**	[ ]	Very severe symptoms; child is very distressed; likely to be confined to bed; unable to go to school/preschool; may be in hospital but child is not entirely dependent on others.
**Needs nursing supervision**	[ ]	Confined to bed; in hospital; very severe symptoms but stable; needs help with self-care which a child the same age can do without help.
**Needs constant supervision**	[ ]	High (24 hrs) medical dependence e.g. In intensive care unit; life-threatening symptoms.

[1] Dosseter DR, Liddle JL, Mellis CM. Measuring health outcome in paediatrics: development of the RAHC measure of function. *J Paediatr. Child Health*. 1996; 32(6):519-24.

HEALTH FUNCTION – (for children 5 years old or more)

Please complete this section if your child is **5 years old or more only**.

If your child is 4 years old or younger, please **Go to Question 43 on page 7**

For each of the next 15 questions, please select ONE answer that best describes your child's level of ability or disability during the past 2 weeks. Although a few questions are similar, please excuse the overlap and answer each question independently.

28. Which ONE of the following best describes your child's ability, during the past 2 weeks, to see well enough to read ordinary newsprint?

- ☐ a. Able to see well enough without glasses or contact lenses.
- ☐ b. Able to see well enough with glasses or contact lenses.
- ☐ c. Unable to see well enough even with glasses or contact lenses.
- ☐ d. Unable to see at all.

29. Which ONE of the following best describes your child's ability, during the past 2 weeks, to see well enough to recognize a friend on the other side of the street?

- ☐ a. Able to see well enough without glasses or contact lenses.
- ☐ b. Able to see well enough with glasses or contact lenses.
- ☐ c. Unable to see well enough even with glasses or contact lenses.
- ☐ d. Unable to see at all.

30. Which ONE of the following best describes your child's ability, during the past 2 weeks, to hear what was said in a group conversation with at least three other people?

- ☐ a. Able to hear what was said with out a hearing aid.
- ☐ b. Able to hear what was said with a hearing aid.
- ☐ c. Unable to hear what was said even with a hearing aid.
- ☐ d. Unable to hear what was said, but did not wear a hearing aid
- ☐ e. Unable to hear at all.

31. Which ONE of the following best describes your child's ability, during the past 2 weeks, to hear what was said in a conversation with one other person in a quiet room?

- ☐ a. Able to hear what was said without a hearing aid.
- ☐ b. Able to hear what was said with a hearing aid.
- ☐ c. Unable to hear what was said even with a hearing aid.
- ☐ d. Unable to hear what was said, but did not wear a hearing aid.
- ☐ e. Unable to hear at all.

32. Which ONE of the following best describes your child's ability, during the past 2 weeks, to be understood when speaking his/her own language with people who do not know the child?

- ☐ a. Able to be understood completely.
- ☐ b. Able to be understood partially.
- ☐ c. Unable to be understood.
- ☐ d. Unable to speak at all.

33. Which ONE of the following best describes your child's ability, during the past 2 weeks, to be understood when speaking with people who know the child well?

- ☐ a. Able to be understood completely
- ☐ b. Able to be understood partially
- ☐ c. Unable to be understood
- ☐ d. Unable to speak at all

34. Which ONE of the following best describes your child's feelings during the past 2 weeks?

- ☐ a. Happy and interested in life
- ☐ b. Somewhat happy
- ☐ c. Somewhat unhappy
- ☐ d. Very unhappy
- ☐ e. So unhappy that life was not worthwhile

Confidential

35. Which ONE of the following best describes the pain and discomfort your child has experienced during the past 2 weeks?

- ☐ a. Free of pain or discomfort.
- ☐ b. Mild or moderate pain or discomfort that prevented no activities.
- ☐ c. Moderate pain or discomfort that prevented some activities.
- ☐ d. Moderate to severe pain or discomfort that prevented some activities.
- ☐ e. Severe pain or discomfort that prevented most activities.

36. Which ONE of the following best describes your child's ability, during the past 2 weeks, to walk?

Note: Walking equipment refers to mechanical supports such as braces, a cane, crutches or a walker.

- ☐ a. Able to walk around the neighborhood without difficulty, and without walking equipment.
- ☐ b. Able to walk around the neighborhood with difficulty, but did not require walking equipment or the help of another person.
- ☐ c. Able to walk around the neighborhood with walking equipment, but without the help of another person.
- ☐ d. Able to walk only short distances with walking equipment, and required a wheelchair to get around the neighbourhood.
- ☐ e. Unable to walk alone, even with walking equipment. Able to walk short distances with the help of another person, and required a wheelchair to get around the neighbourhood.
- ☐ f. Unable to walk at all.

37. Which ONE of the following best describes your child's ability, during the past 2 weeks, to use his/her hands and fingers?

(**Note:** Special tools refers to hooks for buttoning clothes, gripping devices for opening jars or lifting small items, and other devices to compensate for limitations of hands or fingers.

- ☐ a. Full use of two hands and ten fingers.
- ☐ b. Limitations in the use of hands or fingers, but did not require special tools or the help of another person.
- ☐ c. Limitations in the use of hands or fingers, independent with use of special tools (did not require the help of another person).
- ☐ d. Limitations in the use of hands or fingers, required the help of another person for some tasks (not independent even with use of special tools).
- ☐ e. Limitations in the use of hands or fingers, required the help of another person for most tasks (not independent even with use of special tools).
- ☐ f. Limitations in the use of hands or fingers, required the help of another person for all tasks (not independent even with use of special tools).

38. Which ONE of the following best describes your child's ability, during the past 2 weeks, to remember things?

- ☐ a. Able to remember most things.
- ☐ b. Somewhat forgetful.
- ☐ c. Very forgetful.
- ☐ d. Unable to remember anything at all.

39. Which ONE of the following best describes your child's ability, during the past 2 weeks, to think and solve day to day problems?

- ☐ a. Able to think clearly and solve day to day problems.
- ☐ b. Had a little difficulty when trying to think and solve day to day problems.
- ☐ c. Had some difficulty when trying to think and solve day to day problems.
- ☐ d. Had great difficulty when trying to think and solve day to day problems.
- ☐ e. Unable to think or solve day to day problems.

40. Which ONE of the following best describes your child's ability, during the past 2 weeks, to perform basic activities?

- ☐ a. Eat, bathe, dress and use the toilet normally.
- ☐ b. Eat, bathe, dress or use the toilet independently with difficulty.
- ☐ c. Required mechanical equipment to eat, bathe, dress or use the toilet independently.
- ☐ d. Required the help of another person to eat, bathe, dress or use the toilet.

<u>Confidential</u>

41. Which <u>ONE</u> of the following best describes your child's feelings during the <u>past 2 weeks</u>?

☐ a. Generally happy and free from worry.
☐ b. Occasionally fretful, angry, irritable, anxious or depressed.
☐ c. Often fretful, angry, irritable, anxious or depressed.
☐ d. Almost always fretful angry, irritable, anxious or depressed.
☐ e. Extremely fretful angry, irritable, anxious or depressed; to the point of needing professional help.

42. Which <u>ONE</u> of the following best describes the pain or discomfort your child has experienced during the <u>past 2 weeks</u>?

☐ a. Free of pain and discomfort.
☐ b. Occasional pain or discomfort. Discomfort relieved by non-prescription drugs or self-control activity without disruption of normal activities.
☐ c. Frequent pain or discomfort. Discomfort relieved by oral medicines with occasional disruption of normal activities.
☐ d. Frequent pain or discomfort; frequent disruption of normal activities. Discomfort required prescription narcotics for relief.
☐ e. Severe pain or discomfort. Pain not relieved by drugs and constantly disrupted normal activities.

Health Utilities Index Mark 2 and Mark 3 (HUI 2/3) 15-item Questionnaire
©Health Utilities Inc., Dundas ON Canada

HEALTH AND FUNCTION

43. Please indicate if your child currently uses any of the following to help their hearing or speech:

☐ Cochlear implant ☐ Grommets ☐ Sign language
☐ Other (specify)______________________________

44. Please indicate if your child uses any of the following equipment for movement:

☐ Wheelchair ☐ Crutches ☐ Canes ☐ Leg/arm braces
☐ Hand rails ☐ Other (Specify) ______________________________

45. Has your child ever had surgery to help with their movement, eyesight, hearing or speech?

☐ Yes, ☐No

If yes*, please specify;*______________________________

46. Over the last month has your child experienced pain related to their disease?

☐ Yes ☐ No ☐ Don't know

47. If your child experiences pain related to their disease, please indicate methods currently used for pain relief:

☐ Medications (please specify drugs) ______________________________
☐ Physiotherapy ☐ Massage ☐ Occupational therapy

☐ Other (e.g. Music therapy, etc):______________________________

48. Is there any additional equipment, medications or services that have been recommended to you related to the care of your child that you have NOT been able to access/use?

☐ Yes ☐ No ☐ Don't know

If yes, please specify what has been recommended and why you have not been able to access or use it:

____

____

Confidential

TREATMENT

49. Are you aware of specific medicines for your child's condition?

☐ Yes ☐ No ☐ No effective medicines known

If no, please go to question 51

If yes, please specify:____

If yes**,** is your child being treated with this medicine(s)?

☐ Yes ☐ No

If yes, how do you access this medicine? (e.g. clinical trial, orphan drug program, etc)

____

If yes, do you access this medicine(s) in

☐ Australia ☐ Overseas, please specify where: ______________________________

50. Have there been barriers to accessing these medicines for your child?

☐ Yes ☐ No

If yes, please specify: ____

____

51. Is your child currently using other treatments e.g. special diets or dietary supplements?

☐ Yes ☐ No

If yes*,* please indicate

☐ Dietary supplements ☐ Special Diet
☐ Alternate or complementary medicines, please specify______________________________
☐ Other, please specify____

Confidential

HEALTH SERVICE USE

52. From the list below, please tick all of Specialist Doctors that are involved in your child's care, and estimate how often you visited them ***in the last 12 months in an outpatient or a private clinic (excluding overnight admissions to hospital ward):***

Doctors seen in last 12 months	Number of visits	Number of Visits not covered by Medicare
☐ GP/family doctor	________	____________________
☐ Paediatrician	________	____________________
☐ Cardiologist	________	____________________
☐ Rheumatologist	________	____________________
☐ Dermatologist	________	____________________
☐ Respiratory physician	________	____________________
☐ Geneticist	________	____________________
☐ Eye specialist	________	____________________
☐ Psychiatrist	________	____________________
☐ Neurologist	________	____________________
☐ Pain specialist	________	____________________
☐ Surgeon (Specify type)	________	____________________
☐ Other (Please specify)	________	____________________

53. From the list below, please tick all of the Health Professionals (other than Specialists) that are involved in your child's care, and estimate how often you visited them ***in the last 12 months:***

Other Health Professionals seen in last 12 months	Number of visits	Number of Visits not covered by Medicare
☐ Physiotherapist	________	____________________
☐ Speech Pathologist	________	____________________
☐ Occupational Therapist	________	____________________
☐ Optometrist	________	____________________
☐ Psychologist	________	____________________
☐ Social Worker	________	____________________
☐ Dietician	________	____________________
☐ Genetic Counsellor	________	____________________
☐ Dentist	________	____________________
☐ Specialist Nurse	________	____________________
☐ Other (Please specify)	________	____________________

Confidential

54. Please estimate the number of hospital *admissions* (admitted to a hospital ward) your child had over the past 12 months?________________________________

55. How many hospital *visits* (other than admissions with stay *less than 8 hours* e.g. to emergency or to other hospital clinics) has your child had over the past 12 months?

a) Number of Emergency Presentations ____________________________

b) Total number of visits to hospital clinic or therapist ________________________

56. Does your child attend a specialist disease clinic? (e.g. Rett syndrome, Metabolic Diseases)
(Note: a specialist clinic is a centre offering care for a specific disease or group of diseases)

☐ Yes, what is the name of the clinic? ______________________________

☐ No

57. Are you currently accessing support services (OT, Speech or Physiotherapy) through the Department of Ageing Disability and Home Care?

☐ Yes ☐ No

58. In your opinion**,** do you have adequate access to all the health services your child needs?

☐ Yes ☐ No ☐ Unsure

If No, what health care related needs of your child, do you believe are currently not met?____________________

____

59. What are the barriers you currently experience in visiting Specialist Doctors? (*tick **ALL options** that apply*)

☐ No barriers
☐ Transport
☐ Distance of travel
☐ Care for other siblings
☐ Long waiting period
☐ Personal financial cost
☐ Time off work
☐ Lack of available health services
☐ Lack of referral
☐ Other (please specify)

____

60. What are the barriers you experience in visiting Other Health Professionals? (*tick **ALL options** that apply*):

☐ No barriers
☐ Transport
☐ Distance of travel
☐ Care for other siblings
☐ Long waiting period
☐ Personal financial cost
☐ Time off work
☐ Lack of available health services
☐ Lack of referral
☐ Other (please specify)

____

Confidential

61. Please indicate how strongly you agree/disagree with the following statements about the health care of your child:

Statement	***Strongly Agree***	***Agree***	***Unsure***	***Disagree***	***Strongly Disagree***
(a) The role of a GP should be to help coordinate the diverse health requirements of your child					
(b) Your GP has adequate knowledge of your child's rare disease					
(c) Allied health services (physiotherapist, occupational therapists, etc) involved in the care of your child have adequate knowledge of your child's rare disease					
(d) An electronic record of your child's medical history accessible to doctors and hospitals would be an advantage when dealing with the health system					
(e) A specialised centre housing many multidisciplinary health services (medical consultations, genetic advice, physiotherapy, etc) in one location would improve your experience in accessing health care					
(f) You find acquiring and organising specialised equipment for your child easy to arrange/acquire					
(g) You have a health professional involved in the care of your child who coordinates all aspects of your child's health care needs (case manager/coordinator)					
If you ticked agree or strongly agree, which type of health professional takes on the role of case manager/coordinator? (e.g. specialist,GP,nurse)					

Confidential

IMPACT ON FAMILY

62. Please indicate (x) how strongly you agree/disagree with the following statements:

Statement	*Strongly Agree*	*Agree*	*Disagree*	*Strongly Disagree*
Additional income is needed in order to cover medical expenses				
The illness is causing financial problems for the family				
Time is lost from work because of hospital appointments				
I am cutting down the hours I work to care for my child				
Our family gives up things because of my child's illness				
People in the neighbourhood treat us as special because of my child's illness				
We see family and friends less because of the illness				
I don't have much time left over for other family members after caring for my child				
We have little desire to go out because of my child's illness				
Because of the illness, we are not able to go on holidays away from home				
Sometimes we have to change plans about going out at the last minute because of my child's state				
Sometimes I wonder whether my child should be treated 'specially' or the same as a normal child				
I think about not having more children because of the illness				
Nobody understands the burden I carry				
Travelling to hospital is a strain on me				
Sometimes I feel like we live on a roller coaster: in crisis when my child is acutely ill, ok when things are stable				
It is hard to find a reliable person to take care of my child				
I live from day to day and don't plan for the future				
Fatigue is a problem for me because of my child's illness				
Learning to manage my child's illness has made me feel better about myself				
Because of what we have shared we are a closer family				
My partner and I discuss our child's problems together				
We try to treat our child as if he/she were a normal child				
My relatives have been understanding and helpful with my child				

1.Stein RE, Reissman CK. The development of an impact-on-family scale: preliminary findings. *Med Care*. 1980; 18(4):465-72.

Confidential

FAMILY SUPPORT SERVICES AND INFORMATION NEEDS

63. Have you found an organisation or support group within Australia specific to your child's disease?

☐ Yes ☐ No ***If No***, go to ***question 66*** ☐ Don't know

64. Please name any support groups or organisations you are involved with:

____

65. How did you find the relevant support groups and organisations?

☐ Doctors ☐ Internet ☐ Press/media/advertisements ☐ Other patients and families
☐ Friends ☐ Other (please specify): ________________________________

66. Have you searched for or used a support group or organisation overseas?

☐ Yes ☐ No

If yes, what are your reasons?

☐ No group exists in Australia ☐ Recommended to you
☐ Better services offered (please specify) ________________________________
☐Other (please specify)________________________________

67. Were you made aware of support groups/organisations at the time of diagnosis?

☐ Yes ☐ No ☐ Don't know ☐ My child does not have a diagnosis yet

68. How interested are you in finding and utilising support groups and organisations?

☐ Very interested ☐ Interested
☐ Not really interested ☐ Not at all interested

69. In your opinion**,** should information about support groups/organisations be offered at diagnosis?
☐ Yes ☐ No ☐ Don't know

70. Are you interested in being in contact with other families or patients with the same or similar disease?

☐ Very interested ☐ Interested ☐ Not really interested ☐ Not at all interested

71. Are you currently in contact with other families or patients with the same or similar disease?

☐ Yes ☐ No

If yes, by what means do you communicate? (please tick *ALL* options that apply)

☐ In person ☐ By e-mail ☐ The internet (e.g. Facebook) ☐ By phone
☐ Other (specify): ________________________________

72. Do you believe you have been provided with adequate information about your child's rare disease?

☐ Yes ☐ No ☐ Don't know

73. Where did you get information about your child's rare disease?(*tick **ALL options** that apply)*

☐ GP ☐ Specialist ☐ Internet ☐ Other parents/families
☐ Support groups ☐ Brochures/pamphlets ☐ Other (specify) ________________________________

Confidential

74. How would you prefer to learn about your child's disease? (*Tick **ALL options** that apply*):

☐ From your GP ☐ From a specialist ☐ Other parents/families
☐ Internet ☐ Support groups ☐ Brochures/pamphlets

☐ Other method (please specify):______________________________

75. Have you had the opportunity to attend educational sessions/workshops about your child's rare disease?

☐ Yes ☐ No

If yes, please provide details:______________________________

Who provided the sessions?______________________________

76. ***How often*** have you felt stressed over the past month? (please tick ***ONE*** option)

☐ Never ☐ Rarely ☐ Occasionally ☐ Frequently ☐ Always

77. ***How stressed*** have you felt over the past month? (please tick ***ONE*** option)

☐ Not stressed ☐ A little stressed ☐ A lot stressed ☐ Highly stressed

78. Are you currently receiving psychological support from any of the following (tick ***ALL options*** that apply)

☐ Psychologist ☐ Psychiatrist ☐ Counsellor ☐ Social worker ☐ General practitioner
☐ Other ______________________________

79. If you are ***not receiving*** psychological support would you like access to this type of assistance?
☐ Yes ☐ No

If YES, why have you not been able to access this type of support (please comment)

____

80. Does your child have any brothers or sisters?

☐ Yes, please state how many, their sex and ages______________________________

____

☐ No, ***if no*** please go to **question 83**

81. Please indicate how strongly you agree with the following statements about the affected child's siblings over the last 12 months;

Statement	Strongly agree	Agree	Unsure	Disagree	Strongly disagree
Siblings receive less of my attention					
Siblings take on more responsibility for household chores					
Siblings have less opportunity for play or free time					
Siblings miss out on outings with friends more often					
Siblings have missed school days as a result of child's needs					
Siblings display anger or frustration about the affected child's needs					
I expect siblings to help out with affected child's needs					
I expect the girls to help out more than the boys (*if applicable*)					
I have concerns about siblings' emotional well-being					

82. Please indicate how strongly you agree/disagree with the following statements about your affected child's siblings:

Statement	Strongly agree	Agree	Unsure	Disagree	Strongly disagree
Siblings had to "grow-up" more quickly than others their age					
Siblings are well informed about the child's condition					

FINANCIAL SUPPORT

83. Do you have private health insurance?

☐ Yes ☐ No

84. Are you receiving financial assistance from government-based programs (e.g. Carer's allowance, Disability Allowance, etc)?

☐ Yes, please specify____

☐ No

85. Did you have to pay additional expenses not covered by Medicare while seeking a diagnosis (e.g. tests, procedures)?

☐ Yes ☐ No ☐ Don't know

a) ***If yes***, please estimate the approximate amount you spent:

$____

b) ***If yes,*** did this cause financial stress for the family? ☐ Yes ☐ No ☐ DK

Confidential

86. Please estimate out of pocket health related expenses over the past 12 month period (travel, doctor visits, equipment, medications, other). *Please tick the box that best applies:*

☐ <$500 ☐ $500-2000 ☐ $2000-5000 ☐ >$5000

87. Is the financial assistance you receive adequate to cover the cost of caring for your child?

☐ More than adequate ☐ Adequate ☐ Not adequate ☐ Not at all adequate

If not adequate, please specify what is not covered:

☐ Transport ☐ Equipment ☐ Medications ☐ Health services
☐ Other (specify) ______

88. Do you feel that you have been provided with adequate information about financial assistance related to caring for your child?

☐ Yes ☐ No ☐ Don't know

89. Do you feel you have been provided with adequate information about you and your child's legal and social rights (e.g. access to education, etc)?

☐ Yes ☐ No

Please comment:

______
______
______
______

RESEARCH

90. Would you be interested in being kept regularly informed of current research and clinical trials related to your child's rare disease?

☐ Yes ☐ No ☐ Don't know

91. Are you interested in participating in relevant research studies into your child's rare disease?

☐ Yes ☐ No ☐ Don't know

92. Feel free to make any other comments here:

______
______
______

THANK YOU

We appreciate the time you have taken to finish this survey. The information gained from this survey will be valuable in developing better health services and supports for your child, you and your family.

Please return survey to:

Australian Paediatric Surveillance Unit
Kids Research Institute, The Children's Hospital Westmead
Locked Bag 4001, Westmead NSW 2145

Should you have any questions about this study please don't hesitate to contact

Dr Marie Deverell on: 02 9845 3005

Australian Paediatric Surveillance Unit

Level 2, Kids Research Institute | The Children's Hospital at Westmead

L:ocked Bag 4001| Westmead NSW 2145

Tel: 02 9845 3005| Fax: 02 9845 3082

Email: apsu@chw.edu.au |Website: www.apsu.org.au
